# A low pKa ligand inhibits cancer-associated pain in mice by activating peripheral mu-opioid receptors

**DOI:** 10.1038/s41598-020-75509-4

**Published:** 2020-10-29

**Authors:** Ana Baamonde, Luis Menéndez, Sara González-Rodríguez, Ana Lastra, Viola Seitz, Christoph Stein, Halina Machelska

**Affiliations:** 1 Laboratorio de Farmacología, Facultad de Medicina, Instituto Universitario de Oncología del Principado de Asturias (IUOPA), Instituto de Investigación Sanitaria del Principado de Asturias (ISPA), Universidad de Oviedo, C/Julián Clavería 6, 33006 Oviedo, Asturias Spain; 2 Department of Experimental Anesthesiology, Charité-Universitätsmedizin Berlin, Campus Benjamin Franklin, Hindenburgdamm 30, 12203 Berlin, Germany; 3 Present Address: Institute of Biochemistry and Biology, University of Potsdam, Karl-Liebknecht-Str. 24-25, 14474 Potsdam, Germany

**Keywords:** Bone cancer, Chronic pain

## Abstract

The newly designed fentanyl derivative [( ±)-N-(3-fluoro-1-phenethylpiperidine-4-yl)-N-phenyl propionamide] (NFEPP) was recently shown to produce analgesia selectively via peripheral mu-opioid receptors (MOR) at acidic pH in rat inflamed tissues. Here, we examined the pH-dependency of NFEPP binding to brain MOR and its effects on bone cancer-induced pain in mice. The IC_50_ of NFEPP to displace bound [^3^H]-DAMGO was significantly higher compared to fentanyl at pH 7.4, but no differences were observed at pH 5.5 or 6.5. Intravenous NFEPP (30–100 nmol/kg) or fentanyl (17–30 nmol/kg) inhibited heat hyperalgesia in mice inoculated with B16-F10 melanoma cells. The peripherally-restricted opioid receptor antagonist naloxone-methiodide reversed the effect of NFEPP (100 nmol/kg), but not of fentanyl (30 nmol/kg). The antihyperalgesic effect of NFEPP was abolished by a selective MOR- (cyprodime), but not delta- (naltrindole) or kappa- (nor-binaltorphimine) receptor antagonists. Ten-fold higher doses of NFEPP than fentanyl induced maximal antinociception in mice without tumors, which was reversed by the non-restricted antagonist naloxone, but not by naloxone-methiodide. NFEPP also reduced heat hyperalgesia produced by fibrosarcoma- (NCTC 2472) or prostate cancer-derived (RM1) cells. These data demonstrate the increased affinity of NFEPP for murine MOR at low pH, and its ability to inhibit bone cancer-induced hyperalgesia through peripheral MOR. In mice, central opioid receptors may be activated by ten-fold higher doses of NFEPP.

## Introduction

A large number of painful syndromes are driven by peripheral sensory neurons^1,2^ and are typically accompanied by inflammation with tissue acidosis^3,4^. A prominent example is pain associated with tumors, which exhibit a low extracellular pH^5^. In bone cancer, different factors such as augmented glycolysis due to excessive cell proliferation^6^ or enhanced osteoclast activity may account for the increased acidity and inflammation^7^. pH values as low as 4.5 may be reached at the resorptive microenvironment^8^. The diminished pH is also a cause of bone cancer-induced pain through the activation of transient receptor potential cation channel subfamily V member 1 and acid-sensing ion channel 3 in peripheral sensory neurons^9–11^. Under inflammatory conditions, peripheral opioid receptors and their signaling pathways are upregulated and can mediate relevant opioid analgesia in animals and humans^12,13^. We have recently shown in rats that the agonist ( ±)-*N*-(3-fluoro-1-phenethylpiperidine-4-yl)-*N*-phenylpropionamide (NFEPP), which was designed to selectively activate mu-opioid receptors (MOR) at low pH, ameliorates inflammatory, neuropathic and abdominal pain, but does not elicit side effects typically mediated by MOR exposed to physiological pH in the intestinal myenteric plexus or central nervous system (CNS)^14–16^. It appears that a ligand’s pKa should be close to the pH of damaged tissue to obtain antinociception without adverse effects such as respiratory depression, sedation, reward and constipation^17,18^.

Here, we explore the pH-dependency of NFEPP binding to mouse brain MOR and assess the antihyperalgesic effects of NFEPP in murine models of bone cancer-induced pain. To this end, we tested the effects of NFEPP on pain in mice intratibially inoculated with melanoma B16-F10 cells. This cell line induces mixed osteoblastic/osteoclastic activity that causes bone osteolysis and extracellular acidity^19^. Because the analgesic efficacy of some compounds can depend on the type of tumor cells^20^, we also evaluated mice treated with fibrosarcoma NCTC 2472 cells or RM1 cells derived from prostate carcinoma.

## Results

### Binding of NFEPP to brain MOR is increased at low pH

**Figure 1:**
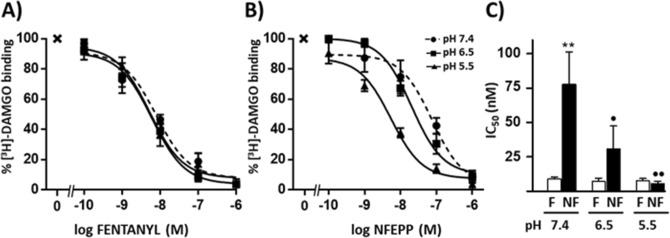
Competition of fentanyl and NFEPP with specific [^3^H]-DAMGO (4 nM) binding in mouse brain membranes at pH 7.4, 6.5 and 5.5. Concentration–response effects of (**A**) fentanyl and (**B**) NFEPP, where x represents the total specific binding in the absence of fentanyl or NFEPP. (**C**) IC_50_ values of fentanyl (F) and NFEPP (NF). Means ± SEM (n = 6 in pH 7.4 groups, n = 5 per group for other pH values) are represented. **P < 0.01, compared to fentanyl at the same pH; ^●^*P* < 0.05, ^●●^*P* < 0.01 compared to NF at pH 7.4, two-way ANOVA and Tukey’s test.

Competition studies were performed in the presence of a fixed concentration of selective MOR ligand [^3^H]-DAMGO ([D-Ala^2^,N-Me-Phe^4^,Gly^5^-ol]-enkephalin; 4 nM) at different pH values. The binding of [^3^H]-DAMGO to brain membranes was dose-dependently inhibited by fentanyl (Fig. 1A) and NFEPP (Fig. 1B) between 10^–10^ and 10^–6^ M. Whereas the concentrations of fentanyl that inhibited [^3^H]-DAMGO binding by 50% (inhibitory concentration 50; IC_50_) were similar at all investigated pH values (7.4, 6.5, 5.5), the corresponding IC_50_ values of NFEPP decreased progressively at acidic pH (Fig. 1C; drug: F_1,26_ = 8.12, *P* = 0.009; pH: F_2,26_ = 4.46, *P* < 0.022; drug x pH: F_2,26_ = 4.04, *P* = 0.03). At physiologic pH (7.4), NFEPP’s IC_50_ was significantly (one order of magnitude) higher than that of fentanyl (*P* < 0.01). In contrast, the IC_50_ values of the both drugs were similar at pH 6.5 (*P* = 0.81) and pH 5.5 (*P* > 0.99) (Fig. 1C).

### Fentanyl and high doses of NFEPP produce antinociception in mice without tumors

**Figure 2:**
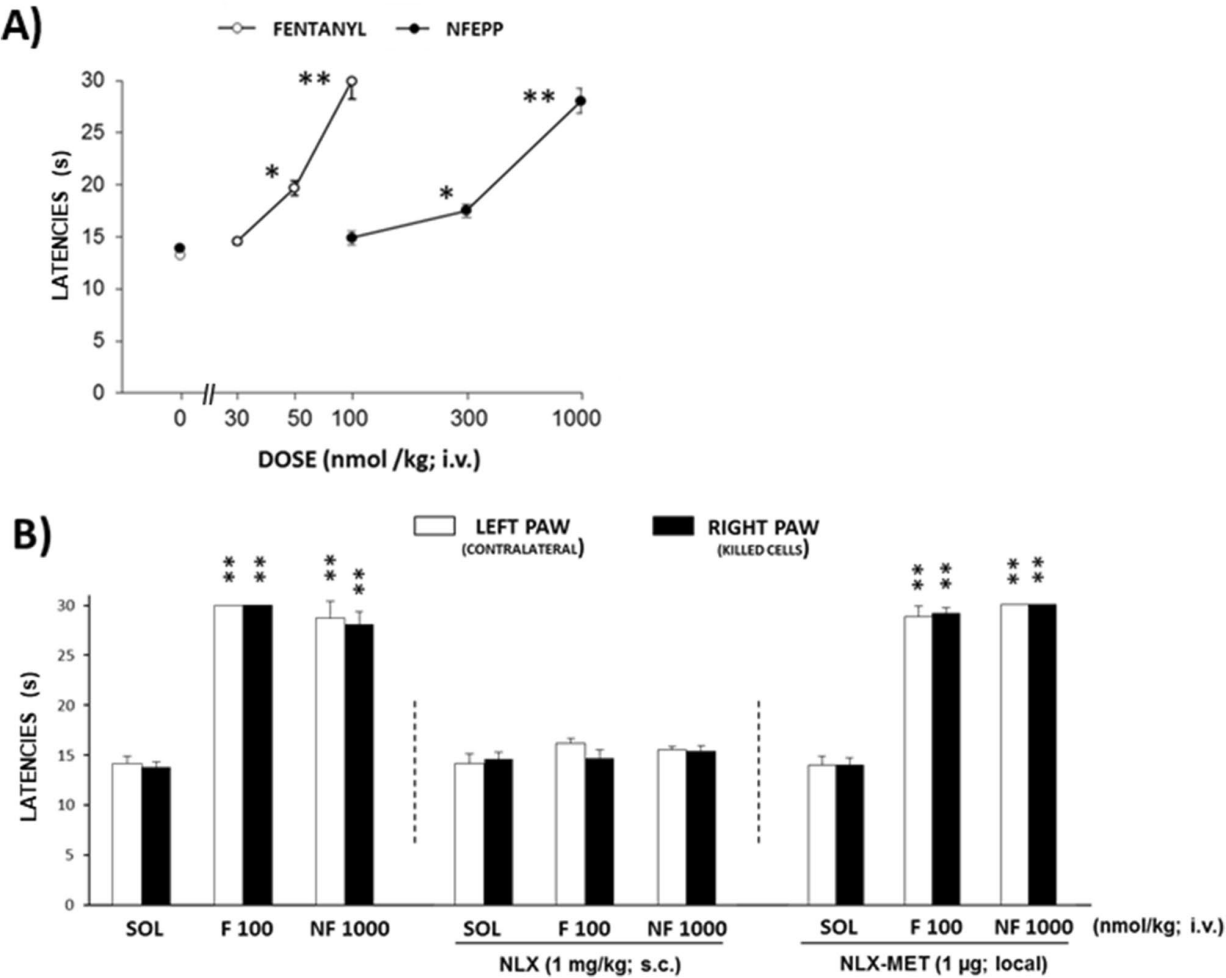
Effects of i.v. fentanyl and NFEPP on heat hyperalgesia in mice inoculated with killed B16-F10 cells. (**A**) Dose–response effects of fentanyl (30–100 nmol/kg) and NFEPP (100–1000 nmol/kg) on ipsilateral paw withdrawal latencies (n = 5 per group). *P < 0.05, **P < 0.01 compared to solvent (0 nmol/kg)-treated mice, one-way ANOVA and Dunnett’s *t* test. (**B**) Systemic naloxone (NLX; 1 mg/kg s.c. at the neck) but not the local injection of naloxone-methiodide (NLX-MET; 1 µg s.c. in the calf) into the inoculated limb inhibited the antinociceptive effect of fentanyl (F) and NFEPP (NF) (n = 7 in F + NLX-MET group, n = 5 per group for other treatments). **P < 0.01 compared to the corresponding paw of the respective solvent (SOL)-treated mice, two-way ANOVA and Tukey’s test. All data are means ± SEM. For clarity, in the graphs a maximal number of two symbols of significancy are represented (the exact values are stated in the results section).

Mice intratibially inoculated with killed B16-F10 melanoma cells do not develop tumors and were used as controls. One week after inoculation with these cells^21^, the intravenous (i.v.) administration of fentanyl (30–100 nmol/kg) or NFEPP (100–1000 nmol/kg) evoked a dose-dependent antinociceptive response in the inoculated limbs (Fig. 2A; fentanyl: F_3,16_ = 54.06, *P* < 0.0001; NFEPP: F_3,16_ = 69.27, *P* < 0.0001). NFEPP produced antinociception at doses of 300–1000 nmol/kg i.v., i.e. at about 6–tenfold higher doses compared to fentanyl (Fig. 2A). Similar effects were observed in the contralateral limbs (data not shown).

To analyze the involvement of central or peripheral opioid receptors, we used non-selective opioid receptor antagonists, naloxone hydrochloride (naloxone) and naloxone-methiodide (NLX-MET). Naloxone acts at both central and peripheral opioid receptors, whereas NLX-MET acts at peripheral opioid receptors, since it does not readily cross the blood–brain barrier^22–25^. Naloxone (1 mg/kg) injected subcutaneously (s.c.) at the neck completely suppressed the antinociceptive effects evoked by fentanyl (100 nmol/kg i.v.) or NFEPP (1000 nmol/kg i.v.) (*P* > 0.99, naloxone with solvent vs. naloxone with fentanyl or NFEPP). In contrast, NLX-MET (1 µg) injected s.c. in the calf (over the tibia site inoculated with killed B16-F10 cells) did not modify the effects of either agonist (*P* < 0.0001, NLX-MET with solvent vs. NLX-MET with fentanyl or NFEPP; Fig. 2B).

### Fentanyl acting centrally and NFEPP acting at peripheral MOR inhibit hyperalgesia in tumor-bearing mice

**Figure 3:**
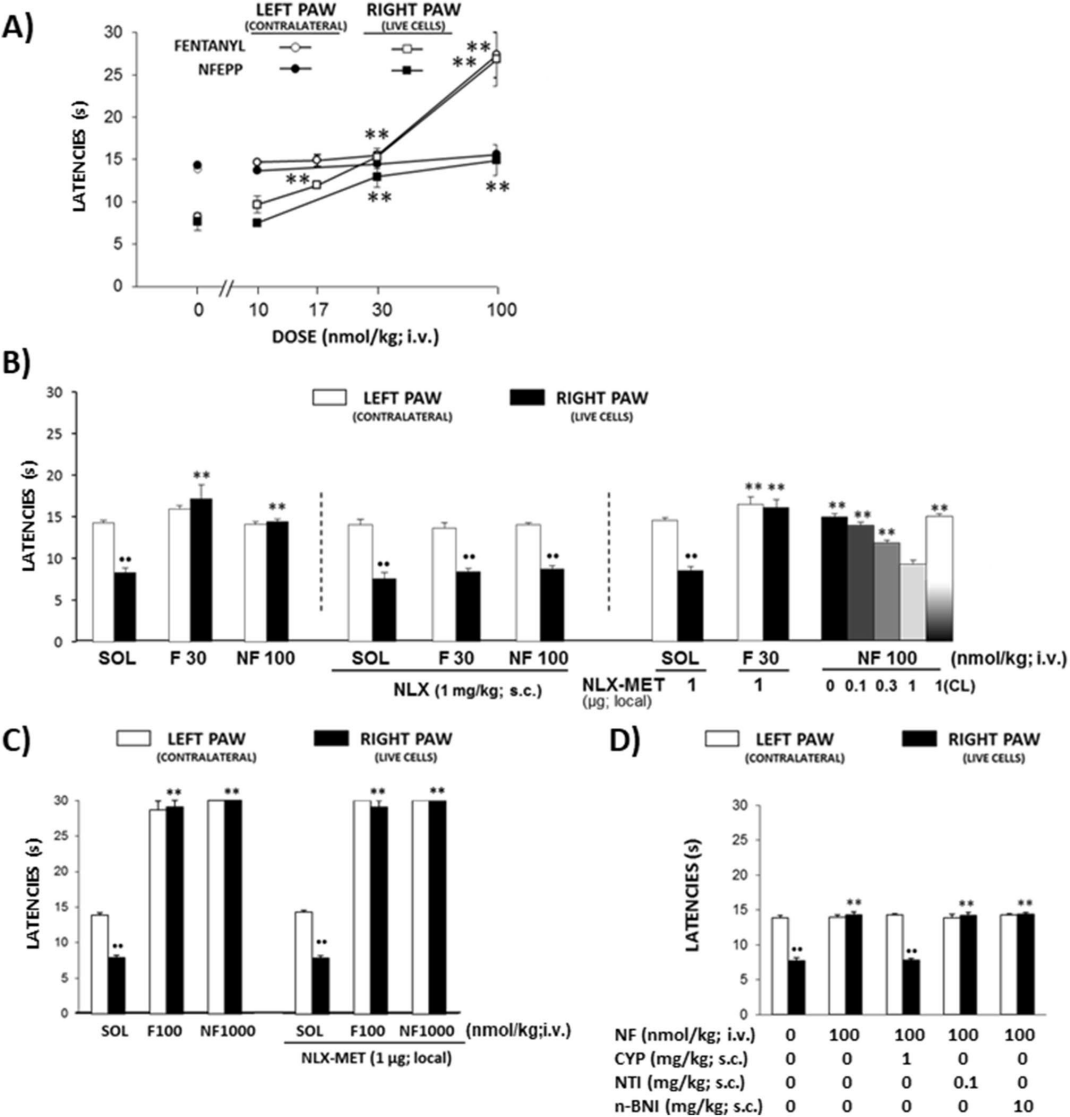
Effects of i.v. fentanyl and NFEPP on heat hyperalgesia in mice inoculated with live B16-F10 cells. (**A**) Dose–response effects of fentanyl and NFEPP (both at 10–100 nmol/kg) (n = 6 in NFEPP 30 nmol/kg group, n = 5 per group for other treatments). (**B**) Systemic naloxone (NLX; 1 mg/kg s.c. at the neck), but not peritumoral naloxone-methiodide (NLX-MET; 1 µg local), inhibited the effect of i.v. fentanyl (F; 30 nmol/kg). Both NLX (1 mg/kg) and NLX-MET (0.1–1 µg) reversed the effect of NFEPP (NF; 100 nmol/kg). NLX-MET (1 µg local) had no effect when injected into non-inoculated, contralateral limb (CL) (n = 6 in solvent [SOL] group, n = 8 in F + NLX-MET group, n = 5 per group for other treatments). (**C**) Peritumoral NLX-MET (1 µg local) did not modify antinociceptive effects evoked by high doses of fentanyl (F; 100 nmol/kg) or NFEPP (NF; 1000 nmol/kg) (n = 6 in SOL + NLX-MET and F + NLX-MET groups, n = 5 per group for other treatments). (**D**) Cyprodime (CYP; 1 mg/kg), but not naltrindole (NTI; 0.1 mg/kg) or nor-binaltorphimine (n-BNI; 10 mg/kg), injected s.c. at the neck, inhibited the effects of NF (100 nmol/kg) (n = 5 per group). In (**A**,**B**), ^●●^*P* < 0.01 compared to the corresponding left paw, unpaired Student’s *t* test; **P < 0.01 compared to solvent (SOL)-treated mice, one-way ANOVA and Dunnett’s *t* test. In (**C**,**D**), ^●●^*P* < 0.01 compared to the corresponding left paw, unpaired Student’s *t* test; **P < 0.01 compared to the corresponding paw in solvent (SOL)-treated mice, two-way ANOVA and Tukey’s test. All data are means ± SEM. For clarity, in the graphs a maximal number of two symbols of significancy are represented (the exact values are stated in the results section).

The intratibial inoculation with live B16-F10 cells provoked the development of tumors leading to unilateral thermal hyperalgesia 1 week later, as previously described^21^. Thus, withdrawal latencies in tumor-inoculated limbs were significantly lower than in contralateral, non-tumoral limbs (*P* < 0.001; Fig. 3A). The i.v. administration of fentanyl (10–100 nmol/kg) dose-dependently inhibited hyperalgesia in the ipsilateral, tumor-bearing limb (F_4,20_ = 21.87, *P* < 0.0001). At a dose of 100 nmol/kg i.v., fentanyl exerted similar (almost maximal) increases in withdrawal latencies in both tumor-bearing and contralateral, tumor-free limbs (*P* < 0.0001 vs. solvent; Fig. 3A). In contrast, only the latencies measured in tumor-bearing limbs were dose-dependently elevated after i.v. NFEPP (10–100 nmol/kg) (F_3,16_ = 9.083, *P* = 0.001) and no effects were observed in contralateral, tumor-free limbs (F_3,17_ = 2.101, *P* = 0.138) (Fig. 3A).

Systemic naloxone (1 mg/kg s.c. at the neck) completely reversed the antihyperalgesic effects induced by both fentanyl (30 nmol/kg i.v.) and NFEPP (100 nmol/kg i.v.) in inoculated limbs (*P* > 0.99, naloxone with solvent vs. naloxone with fentanyl or NFEPP; Fig. 3B). The effects of NLX-MET were assessed against two different doses of the agonists: an antihyperalgesic dose, which restores latencies up to values measured in healthy mice, and an antinociceptive dose that produces an increase in withdrawal latencies above those measured in healthy mice, up to cut-off values. The effect evoked by an antihyperalgesic dose of fentanyl (30 nmol/kg i.v.) was not modified by the peritumoral (s.c. over tibial tumoral mass) administration of NLX-MET (1 µg) (*P* = 0.954). In contrast, the antihyperalgesic effect evoked by 100 nmol/kg (i.v.) NFEPP was dose-dependently inhibited by NLX-MET (0.1–1 µg) applied peritumorally (F_4,20_ = 36.65, *P* < 0. 0001), but not contralaterally (1 µg s.c. in the calf) (*P* = 0.9998) (Fig. 3B). Besides, the bilateral antinociceptive effects evoked by high doses of either fentanyl (100 nmol/kg i.v.) or NFEPP (1000 nmol/kg i.v.) were unaffected by the peritumoral administration of NLX-MET (1 µg) (*P* = 0.801 and *P* > 0.9999, respectively; Fig. 3C).

Systemic (s.c. at the neck) administration of the MOR selective antagonist cyprodime (1 mg/kg s.c.) prevented the antihyperalgesic effect of NFEPP (100 nmol/kg i.v.) (*P* < 0.0001), whereas the selective antagonists of delta-opioid receptors (DOR) naltrindole (0.1 mg/kg s.c.) or of kappa-opioid receptors (KOR) nor-binaltorphimine (10 mg/kg s.c.) had no effects (*P* = 0.7735 and *P* = 0.9710, respectively; Fig. 3D).

### Effects of NFEPP on hyperalgesia evoked by intratibial inoculation with NCTC 2472 or RM1 cells

**Figure 4:**
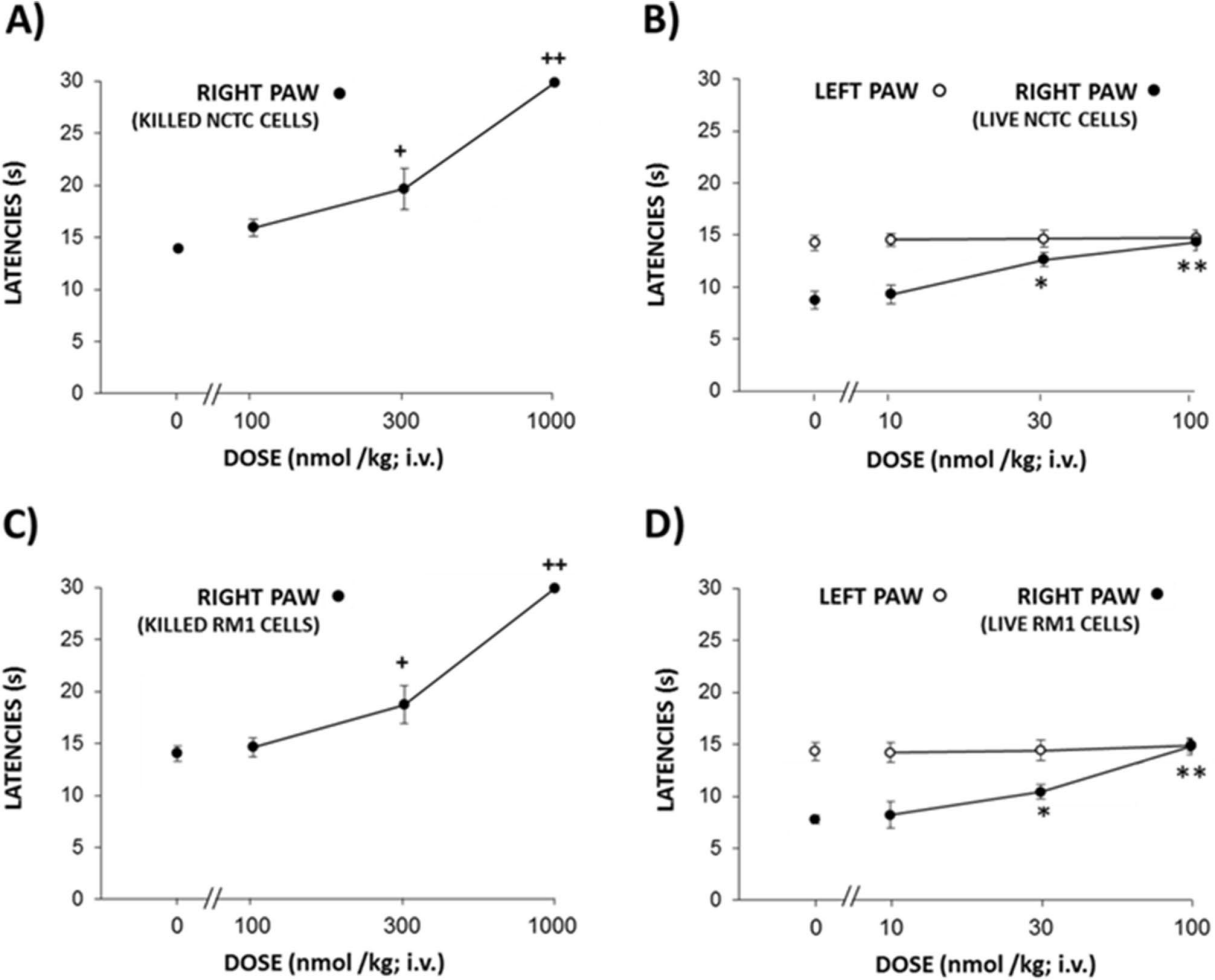
Effects of i.v. NFEPP (10–1000 mg/kg) on heat hyperalgesia in mice inoculated with NCTC 2472 or RM1 cells. Effects in mice inoculated with killed NCTC 2472 cells (n = 8 in 100 nmol/kg group, n = 7 in 300 nmol/kg group, n = 5 per group for other treatments) (**A**) or RM1 cells (n = 5 per group) (**C**), or in tumor-bearing mice inoculated with live NCTC 2472 cells (n = 6 in 30 nmol/kg group, n = 5 per group for other treatments) (**B**) or RM1 cells (n = 6 in 100 nmol/kg group, n = 7 per group for other treatments) (**D**). ^+^*P* < 0.05, ^++^*P* < 0.01, compared to solvent (0 nmol/kg)-treated mice (**A**,**C**); *P < 0.05, **P < 0.01, compared to the solvent-treated mice in the right paw, one-way ANOVA and Dunnett’s *t* test (**B**,**D**). All data are means ± SEM. For clarity, in the graphs a maximal number of two symbols of significancy are represented (the exact values are stated in the results section).

Four weeks after inoculation with killed NCTC 2472 fibrosarcoma cells^26^ (Fig. 4A) or 2 weeks after inoculation with killed RM1 prostate carcinoma cells^27^ (Fig. 4C), which did not induce tumors, high doses of i.v. NFEPP (300–1000 nmol/kg) produced antinociceptive effects in inoculated limbs (F_3,21_ = 25.40, *P* < 0.0001 for NCTC 2472 and F_3,16_ = 178.4, *P* < 0.0001 for RM1 cells). Mice bearing tumors in response to the inoculation of live NCTC 2472 (Fig. 4B) or RM1 cells (Fig. 4D) developed unilateral thermal hyperalgesia that was inhibited by tenfold lower doses of i.v. NFEPP (30–100 nmol/kg) (F_3,17_ = 21.75, *P* < 0.0001 for NCTC 2472 cells and F_3,23_ = 71.23, *P* < 0.0001 for RM1 cells). No effects were observed in contralateral, tumor free limbs at these doses (F_3,17_ = 0.23, *P* = 0.88 for NCTC 2472 cells and F_3, 23_ = 1.802, *P* = 0.17 for RM1 cells) (Fig. 4B,D).

## Discussion

We show here that the fentanyl derivative NFEPP preferentially binds MOR at acidic pH values in mouse brain membranes and that its systemic (i.v.) administration alleviates hyperalgesia in mice bearing bone tumors by acting at peripheral MOR in the injured tissue.

Similar binding characteristics of NFEPP were recently demonstrated in homogenates of MOR-transfected human embryonic kidney (HEK) 293 cells^14^ and rat brain^15^. Here, we examined mouse brain membranes by comparing the effects of NFEPP and fentanyl on the binding of the selective MOR agonist [^3^H]-DAMGO to MOR. Fentanyl displaced [^3^H]-DAMGO binding at nM concentrations, in accordance with previous data^28^. Its affinity for MOR was similar at all pH values studied (7.4, 6.5 and 5.5). In contrast, the IC_50_ values of NFEPP that displaced [^3^H]-DAMGO binding progressively decreased as the acidity of the milieu increased, reflecting an improved affinity of NFEPP to opioid receptors at low pH. Thus, while the IC_50_ values of NFEPP and fentanyl were similar at pH 5.5, the IC_50_ value of NFEPP was substantially greater than that of fentanyl at physiologic pH. These results are in line with those previously reported in MOR-transfected HEK 293 cells^14^ and rat brain membranes^15^.

In coherence with the results of binding experiments, NFEPP showed lower potency than fentanyl to evoke antinociception in uninjured mice. In these mice NFEPP only induced considerable effects at doses tenfold higher (1000 nmol/kg) compared to fentanyl (100 nmol/kg). The efficacy of naloxone and the lack of effect of NLX-MET on the almost maximal and bilateral antinociception in non-injured mice suggest that the antinociceptive effects evoked by fentanyl and by high doses of NFEPP occur through the activation of central opioid receptors. Fentanyl (144 nmol/kg i.v.) produced similar effects when mechanical thresholds were measured by a tail pressure test in healthy mice^24^. The effects of high doses of NFEPP in non-injured mice may be explained by the Henderson-Hasselbalch equation, which predicts that at a concentration of 1000 nmol/kg of NFEPP (pK_a_ = 6.8), about 20% (200 nmol/kg) of the substance will be protonated and capable of activating opioid receptors at pH 7.4. Our experiments suggest that a high enough fraction of this amount may have permeated the blood–brain-barrier to activate opioid receptors in the brain (pH = 7.4) to produce antinociception. In addition, the route of administration has to be considered. Previous data in rats indicated that s.c. injected NFEPP at high dosages did not induce centrally-mediated side effects, but the actions on pain after i.v. injections where not assessed^14,16^. Furthermore, studies on species differences have demonstrated that variations in structure and expression of opioid receptors between mice, rats and humans can have measurable effects on the mediation and magnitude of analgesia^29–32^.

Mice intratibially inoculated with live B16-F10 melanoma cells develop a local bone tumor with mixed osteoblastic/osteolytic histopathological features accompanied by thermal hyperalgesia^21^. In addition, B16-F10 cells can elicit extracellular acidity, probably due to the expression of the a3 isoform of the vacuolar ATPase proton pump in their plasma membrane^19^. Contrasting with the different potency of the two agonists in mice without injury, NFEPP and fentanyl inhibited tumor-induced thermal hyperalgesia at similar doses. Thus, the administration of either ligand at 30 nmol/kg completely inhibited hyperalgesia without modifying withdrawal latencies in contralateral, non-injured limbs. These antihyperalgesic effects were antagonized by systemic naloxone (1 mg/kg). However, peritumoral NLX-MET (1 µg) did not modify the effect induced by fentanyl, supporting that its action is mediated centrally as described in rats with peripheral inflammation^33^ and in tumor-bearing mice inoculated with NCTC 2472 cells^34^. The participation of peripheral opioid receptors in the antinociceptive effects of fentanyl has been reported in mice intraplantarly inoculated with B16-F10 cells when mechanical allodynia or spontaneous licking were measured^24^. Our results may be explained by the rapid access of fentanyl to the CNS by both passive diffusion and carrier-mediated processes^35^. In contrast, the antihyperalgesic effects of NFEPP in doses up to 100 nmol/kg were exclusively mediated peripherally in tumor-bearing mice, since they were completely inhibited by peritumoral NLX-MET. Thus, our results support the notion that the acidity of the tumor environment enables NFEPP to activate local opioid receptors in the injured limb at doses that do not act at opioid receptors in uninjured tissue such as CNS. Although we do not know the exact pH value of the tumor after the intratibial inoculation of B16-F10 cells in our experiments, it has been shown that these cells can induce acidic environments in the metastatic tissue^19^ and that their subcutaneous injection can lower pH values down to 5.8^36^. Finally, the particular bone milieu, where osteoclasts induce bone resorption through proton release, suggests that very low extracellular pH values, such as 4.5, could be reached^8^.

To elucidate the involvement of MOR, DOR and KOR in the inhibition of tumoral hyperalgesia evoked by NFEPP, we tested the effects of the cyprodime, naltrindole and nor-binaltorphimine at doses able to block selectively MOR, DOR and KOR, respectively^37,38^. The efficacy of cyprodime, but not naltrindole and nor-binaltorphimine, to revert the effect of NFEPP indicates that the inhibition of tumor hyperalgesia evoked by NFEPP occurs through the activation of MOR exclusively. Thus, although the chemical structure of NFEPP led to an improved binding affinity at low pH values, its selectivity for MOR seems similar to that of fentanyl, consistent with our initial report^14^.

The efficacy of some drugs to inhibit bone tumor-induced hyperalgesia can depend on the type of neoplastic process^20,39^. Therefore, we also assayed NFEPP’s ability to inhibit thermal hyperalgesia in mice inoculated with NCTC 2472^26^ or RM1 cells^27^. The effects in mice inoculated with killed NCTC 2472 or RM1 cells confirmed that high doses of NFEPP are needed to produce antinociception in mice without tumors. In mice inoculated with live NCTC 2472 or RM1 cells, NFEPP counteracted thermal hyperalgesia with a similar potency as that observed in mice treated with live B16-F10 cells, demonstrating its efficacy in different types of bone tumors. This is coherent with its mechanism of action, since the main variable involved in the analgesic activity of NFEPP seems related to tissue acidification, a common property of bone tumors independent of the cell line used^5^.

In conclusion, the present data demonstrate that NFEPP can inhibit hyperalgesia by acting at peripheral MOR in the acidic tumoral environment in mice. The activation of central opioid receptors may be due to excessively high doses of NFEPP yielding sufficient amounts of protonated compound to activate opioid receptors at normal pH, and/or to species differences in structure and signaling of opioid receptors^29–32^. Together with the inhibition of enkephalin-degrading enzymes^25^, the blockade of P2X3 receptors^40^ or the stimulation of endothelin-B receptors^41^, the acid-related antihyperalgesic effect induced by NFEPP adds a new approach to counteract tumoral hyperalgesia through the activation of peripheral opioid receptors. In view of current discussions surrounding the predictivity of animal models in pain research^42^, it will be particularly important to examine the effects of NFEPP in humans.

## Methods

### Animals

Experiments were performed in 5–6 weeks old (26–33 g) C57BL/6 and C3H/He male mice bred in the Animalario de la Universidad de Oviedo (Reg. 33044 13A). Mice were housed in groups of 8–10 per cage, lined with a sawdust bedding, and maintained at 21 °C and 35% humidity on a 12-h dark–light cycle, with free access to food and water. All experimental procedures were approved by the Comité Ético de Experimentación Animal de la Universidad de Oviedo (Asturias, Spain) and performed according to the guidelines of European Communities Council Directive (2010/63/EU) for animal experiments. Each animal was used only once.

### Compounds and their administration

NFEPP was synthesized by a contractor (ASCA GmbH, Berlin, Germany) according to our computer-assisted design^14^. It was dissolved in dimethyl-sulfoxide (DMSO; 4 mg/ml) at a maximal concentration of 1.75%. Fentanyl citrate (Sigma) was dissolved in 0.9% NaCl. In order to compare the effects produced by these drugs, the amounts of NFEPP or fentanyl administered are expressed on a molar basis, since fentanyl represents only 63.65% of the entire molecular weight of the citrate salt. NFEPP (10–1000 nmol/kg corresponding to 3.6–355 µg/kg) and fentanyl (10–100 nmol/kg corresponding to 5.3–53 µg/kg of fentanyl citrate) were administered i.v. in a final volume of 5 ml/kg 10 min before testing. The non-selective opioid receptor antagonist naloxone (1 mg/kg; Tocris), the MOR selective antagonist cyprodime hydrobromide (1 mg/kg; Sigma) and the respective DOR and KOR antagonists naltrindole hydrochloride (0.1 mg/kg; Tocris) and nor-binaltorphimine dihydrochloride (10 mg/kg; Tocris) were dissolved in 0.9% NaCl and administered s.c. into a skin fold of the dorsal side of the neck in a final volume of 10 ml/kg 30 min before testing. Naloxone-methiodide (NLX-MET 0.1–1 µg; Sigma), a non-selective, peripherally-restricted opioid receptor antagonist^22–25^, was dissolved in 0.9% NaCl and injected s.c. (100 µl) over the tibial tumoral mass (peritumoral administration) 10 min before the test. The administration in limbs without tumor was performed s.c. in the calf, the region corresponding to the peritumoral administration in tumor-bearing mice. Control mice received the respective solvents. The selective MOR ligand [^3^H]-DAMGO (specific activity 49.2 Ci/mmol, Perkin-Elmer) was used in binding assays.

### Binding assays

Untreated C57BL/6 mice were killed by decapitation under light isoflurane anesthesia. Brains without cerebella were removed and homogenized in 5 ml of ice-cold 50 mM TrisHCl (subsequently referred to as buffer) using a PT 3100 Kinematica Polytron coupled to a PT-DA 3012/2 S Homogenizer Generator (Littau, Switzerland) for 20 s, set at 5000 rpm. The homogenate was centrifuged (Optima L-90 K Ultracentrifuge, Beckman) at 42,000 g for 20 min at 4 °C. The pellet was resuspended in ice-cold buffer (pH 7.4), vortexed and centrifuged as before. The final pellet obtained from a single brain homogenate was suspended again in 6 ml buffer at pH 7.4, briefly homogenized, divided into 3 samples of 2 ml and further centrifuged. The resulting pellets were resuspended in ice-cold buffers at different pH values (7.4, 6.5 and 5.5) up to a protein concentration of 2 mg/ml^43^. For competition studies, 50 µl of crude membrane suspensions were incubated in agitation and protected from light at room temperature for 40 min with 4 nM [^3^H]-DAMGO together with buffer only (100% binding of [^3^H]-DAMGO) or with different concentrations of fentanyl (10^–10^–10^–6^ M), NFEPP (10^–10^–10^–6^ M) or naloxone (10^–4^ M) to determine non-specific binding in a final volume of 500 µl. Separate experiments were performed, in which the pH of all reagents, membranes and buffer was either 7.4, 6.5 or 5.5. The binding reaction was terminated by rapid filtration of the mixture through Whatman GF/B filters pre-soaked for 30 min in 0.1% bovine serum albumin incubation buffer. The filters were washed twice with 5 ml ice-cold buffer and transferred to polyethylene counting vials. Three ml of scintillation cocktail Optiphase ‘Hisafe’ 3 (Perkin-Elmer) were added to each vial, which was counted 24 h later by a Wallac 1409 Scintillation Counter (Turku, Finland). All experiments were run in duplicate and repeated 5–6 times. Competition curves were fitted by non-linear regression using the one-site competition fitting option. The total specific binding obtained in the absence of fentanyl or NFEPP was considered the 100% binding and the concentration of compounds resulting in a 50% reduction of [^3^H]-DAMGO binding (IC_50_) was calculated.

### Cell cultures and cell inoculation

B16-F10 melanoma cells (American Type Culture Collection, ATCC) were cultured in Dulbecco’s modified Eagle’s medium (DMEM; Gibco) enriched with 10% fetal calf serum (FCS; Gibco). Once preconfluence was reached, cells were treated with trypsin/EDTA (0.05%/0.02%) and detached. The trypsin/EDTA solution was recovered, neutralized with DMEM, supplemented with 10% FCS and centrifuged at 400*g* for 10 min. The remaining pellets were suspended in PBS^20^.

NCTC 2472 fibrosarcoma cells (ATCC) were cultured in NCTC 135 medium (Sigma) containing 10% horse serum (Sigma), and were passaged weekly according to ATCC guidelines. When cells were confluent, they were detached by scraping, centrifuged at 400*g* for 10 min, and the remaining pellet was suspended in PBS^26^.

RM1 prostate carcinoma cells (kindly donated by Dr. Timothy Thompson, MD Anderson Cancer Center, University of Texas) were cultured in DMEM (low glucose) + glutamine (DMEM + GlutaMAX; Gibco) enriched with 10% FCS (Gibco), HEPES (1 M) (Cellgro), and penicillin (10,000 U/ml)-streptomycin (10 mg/ml) (Biochrom AG). When cells were preconfluent, they were treated with trypsin/EDTA (0.05%/0.02%) and detached. The trypsin/EDTA solution was neutralized with growth medium (1:1) and, after centrifugation at 400*g* for 10 min, the remaining pellet was suspended in PBS^27^.

For surgical procedures, anesthesia was induced by spontaneous inhalation of 3% isoflurane (Isoflo^®^; Esteve) and maintained by 1.5% isoflurane in oxygen through a breathing mask. A suspension of 10^5^ B16-F10 cells in 5 µl PBS was injected into the right tibial medullar cavity of C57BL/6 mice. Next, acrylic glue (Hystoacril^®^; Braun) was applied onto the incised area and surgery was completed with a skin stitch. The same procedure was applied in C3H/He mice receiving 10^5^ NCTC 2472 cells and in C57BL/6 mice inoculated with 10^3^ RM1 cells. Control mice were inoculated with the same number of cells previously killed by shock freezing three times without cryoprotection.

### Unilateral hot plate test

Thermal withdrawal latencies were measured using the unilateral hot plate (IITC Life Science). As described previously^44^, mice were gently restrained and the plantar side of one hind paw was placed on the hot plate maintained at 49.1 ± 0.2 °C. The same procedure was performed on the other hind paw. Withdrawal latencies of each paw were measured twice at two-minute intervals, the mean of two measures was calculated, and a cut-off of 30 s was employed. In order to habituate mice to test environment and to discard abnormal withdrawal reactivity in particular individuals, a measurement of basal latencies was taken in each hind paw at least 5 h before starting experiments. Our methodological approach was to exclude from the assays those mice exhibiting basal latencies in healthy paws above 20 s or not showing reduced withdrawal latencies in tumoral paws. However, since no aberrant responses were detected, no animal was discarded for this reason. The experimenter was blinded to the treatments. Based on previous reports, behavioral measures of thermal hyperalgesia were performed 1 week after the intratibial inoculation of B16-F10 cells^21^, and 4 or 2 weeks after the inoculation of NCTC 2472 or RM1 cells, respectively^26,27^.

### Statistical analysis

Data were analyzed using the GraphPad Prism^®^ version 6.01. All data were normally distributed and of equal variances as assessed by Kolmogorov–Smirnov normality test. In binding experiments, means and standard errors of the means (SEM) of IC_50_ values for each condition (pH value and compound) were calculated. Comparisons between IC_50_ values were performed by two-way analysis of variance (ANOVA) followed by the Tukey’s test. In behavioral studies, the mean and SEM of paw withdrawal latency values were calculated. Comparisons between two groups were made by the unpaired Student’s *t* test for independent data, whereas one-way ANOVA followed by the Dunnett’s *t* test was used to compare effects induced by different doses of compounds in one paw. The effects of different doses of a compound or of different compounds in both paws were assessed by two-way ANOVA followed by the Tukey’s test. Statistical significance was considered at *P* < 0.05.

## Data Availability

All data generated and analyzed during this study are included in this published article. All raw data are available from the corresponding author on reasonable request.
